# Marine Fish Proteins and Peptides for Cosmeceuticals: A Review

**DOI:** 10.3390/md15050143

**Published:** 2017-05-18

**Authors:** Jayachandran Venkatesan, Sukumaran Anil, Se-Kwon Kim, Min Suk Shim

**Affiliations:** 1 Division of Bioengineering, Incheon National University, Incheon 406-772, Korea; venkatjchem@gmail.com; 2 Department of Preventive Dental Sciences, College of Dentistry, Prince Sattam Bin Abdulaziz University, Riyadh, Post Box 153, AIKharj 11942, Saudi Arabia; drsanil@gmail.com; 3 Department of Marine Life Sciences, Korean Maritime and Ocean University, 727 Taejong-ro, Yeongdo-Gu, Busan 49112, Korea

**Keywords:** marine fish, cosmeceuticals, proteins, peptides, hydrolysates, collagen, antioxidant, anti-photoaging

## Abstract

Marine fish provide a rich source of bioactive compounds such as proteins and peptides. The bioactive proteins and peptides derived from marine fish have gained enormous interest in nutraceutical, pharmaceutical, and cosmeceutical industries due to their broad spectrum of bioactivities, including antioxidant, antimicrobial, and anti-aging activities. Recently, the development of cosmeceuticals using marine fish-derived proteins and peptides obtained from chemical or enzymatical hydrolysis of fish processing by-products has increased rapidly owing to their activities in antioxidation and tissue regeneration. Marine fish-derived collagen has been utilized for the development of cosmeceutical products due to its abilities in skin repair and tissue regeneration. Marine fish-derived peptides have also been utilized for various cosmeceutical applications due to their antioxidant, antimicrobial, and matrix metalloproteinase inhibitory activities. In addition, marine fish-derived proteins and hydrolysates demonstrated efficient anti-photoaging activity. The present review highlights and presents an overview of the current status of the isolation and applications of marine fish-derived proteins and peptides. This review also demonstrates that marine fish-derived proteins and peptides have high potential for biocompatible and effective cosmeceuticals.

## 1. Introduction

Oceans cover about 70% of the earth’s surface and are inhabited by a large variety of living organisms. The marine environment serves as an enormous resource that provides abundant bioactive substances in the form of food, cosmeceuticals, and pharmaceutical products. Recently, much attention has been paid to obtaining bioactive proteins and peptides from various marine organisms, including fish, algae, crustaceans, and sponges, for cosmeceutical and pharmaceutical applications [1,2]. Marine bioactive proteins and peptides, depending on their structures and amino acid sequences, exhibit a wide range of biological activities including antioxidant, antimicrobial, anticancer, immunomodulatory, antihypertensive, anticoagulant, and anti-diabetic effects [3,4]. 

Marine fish is mostly used as a source of food for human consumption, which has resulted in several fish processing industries producing fish meat. However, these industries discard huge amounts of waste containing fish skin and bones, which in turn aggravate the problem of environmental pollution. To avoid such issues, by-products generated by seafood processing industries are utilized to isolate bioactive compounds beneficial for human health. This process not only assists in decreasing the pollution but also increases the value of the by-products from fish processing [5,6,7]. Fish processing waste contains significant amounts of useful proteins, which represent a source for bioactive peptide mining. For example, collagen is one of the most abundant proteins that can be extracted from the skin, bones, and scales of fish. Collagen has been extensively utilized for various applications, including cosmeceuticals [8], functional foods [9], tissue engineering [10,11], and anti-diabetic medications [12].

In addition to bioactive proteins, various bioactive peptides can be produced from marine fish via chemical or enzymatical hydrolysis. The peptides, which are present in the inactive form within the protein chains, are activated after their hydrolysis using enzymes, including trypsin, proteinases, chymotrypsin, alcalase, and pepsin [3,13,14,15]. Marine fish waste-derived bioactive peptides have gained tremendous interest in nutraceutical and cosmeceutical industries due to their broad spectrum of bioactivities, including antioxidant, antimicrobial, antihypertensive, calcium-binding, and obesity control properties [3,16]. This review describes various bioactive proteins and peptides, which were identified in marine processing waste with emphasis on their potential bioactivities for cosmeceutical applications. Moreover, it outlines current technologies used in the production and purification of the marine fish-derived proteins and peptides.

## 2. Marine Fish Proteins and Peptides

**Figure 1:**
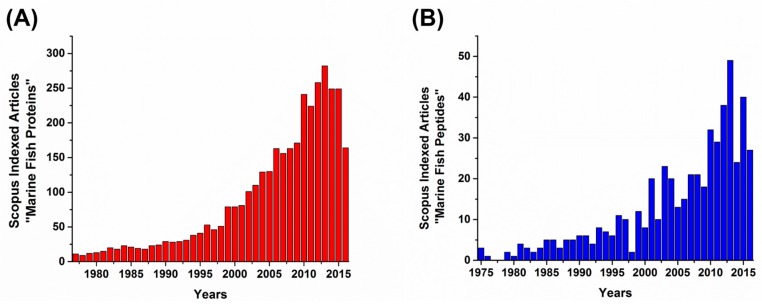
Articles indexed in Scopus with the keywords (**A**) marine fish proteins and (**B**) marine fish peptides. Graph shows the continuous research growth on marine fish proteins and peptides. The bar graph highlights the number of articles indexed in Scopus on “marine fish proteins”, which is greater than that of “marine fish peptides”.

Figure 1 depicts the increasing number of the studies on marine fish-derived proteins and peptides in the last two decades. Marine fish proteins mainly consist of collagen, which has been widely utilized in cosmeceutical areas owing to its moisturizing properties. In addition, it has been extensively studied in pharmaceuticals, nutraceuticals, and food applications. Collagen can be isolated from by-products of fish processing, such as fish bones and fish skin [17,18,19]. 

## 3. Marine Fish-Derived Collagen

Collagen is a main structural protein in connective tissues of skin and bone. It is commonly obtained from bovine and porcine skin. The bovine and porcine collagens have been extensively used for pharmaceutical, cosmeceutical, and nutraceutical purposes. However, the outbreak of certain transmissible diseases such as bovine spongiform encephalopathy and some religious issues associated with the use of bovine proteins hamper their use. Hence, there has been a need to find a suitable alternative to solve these issues, which has led several researchers to turn toward marine sources for the production of collagen. Marine-derived collagen has an ability to scavenge free radicals, and thus can be utilized for skin care products [17,20,21]. Marine-derived collagen has also been widely used as a scaffold for tissue engineering due to its excellent bioactive properties, including biocompatibility, low antigenicity, high biodegradability, and cell growth potential [22,23,24]. There are two types of collagen: fibrillar and nonfibrillar. Marine fish often contain Type I fibrillar collagen in skin and bones [25].

### 3.1. Isolation of Marine Fish-Derived Collagen

**Figure 2:**
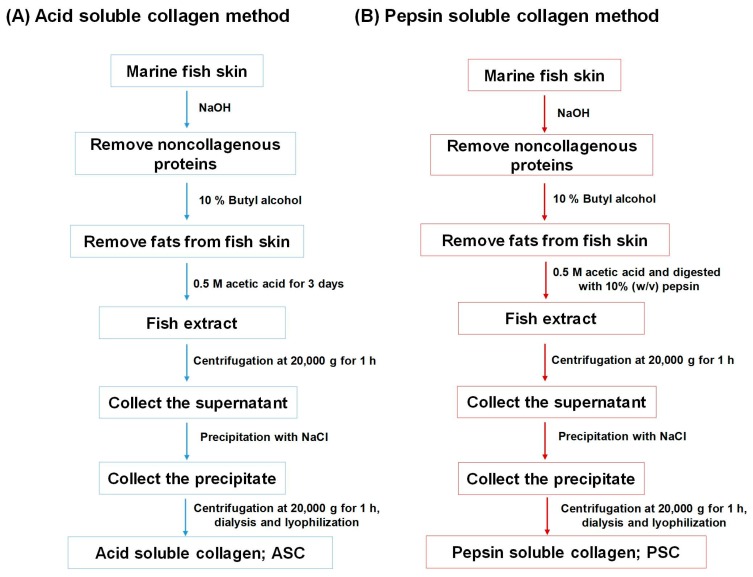
A flowchart for the isolation of collagen from marine fish skin. (**A**) acid-soluble collagen (ASC) method and (**B**) pepsin-soluble collagen (PSC) method.

Although around 75% of the fish weight consists of skin, bones, head, and scales, they are often discarded as by-products by the seafood processing industries [26]. These by-products are a rich source of collagen with a variety of bioactivities. Figure 2 shows the common procedures for isolating collagen from the skin and bones of marine fish [19,26,27]. Acid solubilization and pepsin solubilization are major methods for isolating collagen from various parts of fish species (e.g., skin, bones, and scales). For the acid-soluble collagen (ASC) method, 0.5 M acetic acid is used to digest the fish skin in sufficient time, whereas 10% *w*/*v* pepsin is used for the pepsin-soluble collagen (PSC) method. Table 1 shows a list of some important marine fish species used for collagen isolation. It is observed that the PSC method leads to higher amounts of collagen as compared to the ASC method [19,27,28]. This implies that pepsin in the PSC method is more efficient in digesting skin or bone tissues as compared to acid solution in the ASC method. 

### 3.2. Marine Fish-Derived Collagen in Cosmeceuticals

Marine fish-derived collagen is extensively employed in the development of cosmeceutical products due to its excellent bioactivity toward skin repair and regeneration. The marine fish-derived collagen possesses a higher absorbing capacity than the collagen from animal sources [44]. In addition, marine fish-derived collagen has low odor and improved mechanical strength, prerequisites for cosmetic products [8]. Skin-hydrating and skin-firming effects of cosmetic formulations (cream or serum formulations) using collagen derived from fish were evaluated [45]. The result suggested that serum formulations displayed a better moisturizing effect within a short duration [44,45]. The cream formulations appeared to become more active later, particularly following the repetitive applications. However, a sustained tensor (firming) effect was observed during the treatment using both the lotion and the cream [45]. 

### 3.3. Marine Fish-Derived Collagen in Wound Healing and Tissue Engineering

Tissue-engineered skin substitutes serve as a promising therapeutic agent in replacing the skin lost in wounds such as burns by providing cells, bioactive compounds, bioactive polymers, and proper microenvironments, thereby initiating the wound healing process [46,47,48]. Currently, a main source of collagen is bovine skin and tendons as well as porcine skin, which suffer from drawbacks such as transmission of prions [49]. Therefore, marine organism-derived materials have become initiators or co-initiators of hundreds of promising pharmaceutical and tissue-engineered skin substitutes [50]. Many studies based on marine organism-derived collagen scaffolds for skin tissue regeneration have demonstrated a high potential in clinical applications [51]. In this regard, a composite film comprising salmon milt DNA and salmon collagen showed a remarkable efficacy in wound regeneration [52]. The implantation of the film into a full-thickness wound in the rat dorsal region resulted in tissue regeneration with a morphological appearance similar to that of native rat dermis tissues. In addition, it significantly enhanced the formation of blood capillaries [52]. 

The abundant presence of type I collagen in fish bone tissues has widely increased the applications of collagen-based scaffolds for bone tissue engineering [53,54,55]. Collagen plays an important role in stimulating the differentiation of bone progenitor cells into osteoblasts through interaction with transmembrane α2β1 integrin receptors, and subsequently eliciting cell growth and mineral production [56,57]. The incorporation of glycosaminoglycans (GAGs) into collagen has shown to enhance osteoblastic differentiation of mesenchymal stem cells (MSCs) both in vivo and in vitro [58,59]. 

## 4. Marine Fish-Derived Peptides

Marine fish proteins consist of small peptides, which are often present in the inactive form with a full protein sequence. Enzymatic hydrolysis is frequently used to isolate short and bioactive peptides from marine organisms and seafood waste products. A large amount of histidine-containing dipeptides, carnosine (β-alanylhistidine), and anserine (β-alanyl-1-methylhistidine) are present in tuna, salmon, and eels [60]. Peptides serve as important active ingredients for several pharmaceutical and cosmeceutical applications [4,61,62]. The bioactive peptides are usually made up of 3–20 amino acid residues. Marine fish-derived peptides exhibit various biological activities such as antioxidant, antimicrobial, and angiotensin-I-converting inhibitory activity, as well as cancer metastasis inhibition, and immunostimulant activity [63,64,65]. The most commonly used proteinases for the hydrolysis of fish proteins include alcalase, chymotrypsin, and pepsin [66,67,68].

### 4.1. Isolation of Marine Fish Peptides

**Figure 3:**
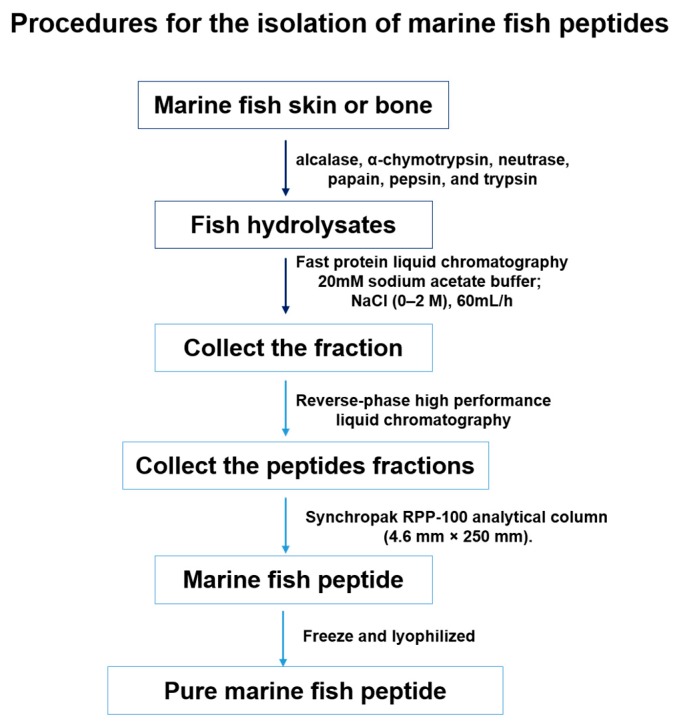
The flowchart showing the common procedures for the isolation and identification of the marine fish-derived peptides through enzymatic hydrolysis methods [70].

Enzymatic hydrolysis is one of the commonly used methods to obtain bioactive peptides. The mechanistic study of the enzymatic hydrolysis of fish proteins is described elsewhere [69]. The general procedures to produce collagen peptides from marine fish skin and bone are shown in Figure 3. Various antioxidant marine fish-derived peptides were obtained through enzymatic hydrolysis methods [70,71]. Different kinds of enzymes (e.g., alcalase, α-chymotrypsin, neutrase, papain, pepsin, and trypsin) were used for the optimized conditional buffer system (Table 2) [70]. The peptides are commonly separated using chromatographic techniques and ultrafiltration membranes. The same group also reported the use of a series of ultrafiltration membranes to separate the peptides [72]. Fast protein liquid chromatography (FPLC) and reverse phase high-pressure liquid chromatography (RP-HPLC) were widely utilized to purify the peptides. 

### 4.2. Biological Activities of Marine Fish Peptides as Cosmeceuticals

An increasing interest in health, well-being, and physical appearance has resulted in high demand for various cosmetics. Recently, a combination of cosmetics with pharmaceuticals and marine-derived biologically active ingredients has become the hallmark of cosmetic industries [15,73,74,75,76]. Antioxidant, anti-inflammatory, reduction of melanin synthesis, tyrosinase inhibition, and matrix metalloproteinase (MMP) inhibitor tests are important in the development of cosmeceuticals against aging and wrinkling of the skin (Table 3). 

### 4.3. Antioxidant Fish Peptides

Antioxidants play an important role in providing protection against oxidative stress. The generation of oxidative stress is attributed to the formation of several reactive oxygen species, including alkyl radicals, hydroxyl radicals, superoxide radicals, peroxide radicals, and singlet oxygen species. In the human body, an imbalance between the free radicals and antioxidants leads to skin damage, inflammation, cancer, and neuron-related diseases [78]. The highly reactive free radicals can easily damage cellular membranes, DNA, proteins, and lipids, and are widely accepted as the primary reason for skin aging [79]. The human body possesses various antioxidant enzymes (e.g., catalase, superoxide dismutase, and glutathione peroxidase) and biomolecules (e.g., vitamin C, vitamin glutathione, and ubiquinone) to control the free radicals inside [79]. In addition, several synthetic products are often used to inhibit free radical activity (e.g., butylated hydroxyanisole (BHA), butylated hydroxytoluene (BHT), tert-butylhydroquinone (TBHQ), and propyl gallate [80]). However, the major drawback of using these antioxidants is the safety concern. Therefore, considerable attention has been diverted to the use of naturally-derived antioxidants [81,82,83,84,85]. Recently, a number of studies have demonstrated that various peptides derived from marine fish serve as effective antioxidants (Table 4) [71,86,87,88,89,90,91,92,93,94,95,96,97,98]. Enzymes for the isolation of antioxidant peptides from the marine fish are also described in Table 4.

Various types of methods have been used to evaluate the antioxidant activity of fish-derived peptides, including the 2,2-diphenyl-1-picrylhydrazyl (DPPH) radical scavenging assay, the 2,2′-azino-bis(3-ethylbenzothiazoline-6-sulphonic acid) (ABTS) radical scavenging assay, hydroxyl radical scavenging activity, Cu^2+^ chelating activity, and Fe^2+^ chelating activity [99,100,101,102,103,104,105].

### 4.4. Antimicrobial Fish Peptides

Antimicrobial peptides possess cationic moieties, which facilitate their interaction with membranes of microbial pathogens [106]. Antimicrobial peptides from marine organisms constitute a new generation of antibiotics. They are currently extensively studied in the development of cosmeceutical products, including lotions, shampoos, and moisture creams. Numerous studies have reported that marine fish-derived peptides can be used as antimicrobial agents, as shown in Table 5 [103,104,105,106]. The enzymes used for the isolation of antimicrobial fish peptides and the microorganisms susceptible to these antimicrobial peptides were listed in Table 5.

### 4.5. Matrix Metalloproteinases Inhibiting Fish Peptides

MMPs are endopeptidases containing zinc metal ion with an ability to degrade extracellular components. MMPs are produced by a variety of cells, including fibroblasts, keratinocytes, mast cells, macrophages, and neutrophils. Six different kinds of MMPs are available, which consist of collagenases, gelatinases, stromelysins, matrilysins, membrane-type MMPs, and other MMPs. The MMPs are categorized into three major functional groups. They include interstitial collagenases with affinities toward collagen types I, II, and III, (MMP-1, -8, and -13, respectively), stromelysins with specificity for laminin, fibronectin, and proteoglycans (MMP-3, -10, and -11, respectively), and gelatinases that effectively cleave type IV and V collagens (MMP-2 and -9) [67].

Wrinkles are a typical symptom of skin aging, and are associated with the reduction in the amount of collagen that dominates the elasticity of the skin dermal tissues. Since collagen fibers and other extracellular matrix are readily degraded by MMPs, formation of wrinkles is closely associated with increased expression of MMPs throughout the skin aging. Therefore, a variety of MMP inhibitors have been utilized to prevent the formation of wrinkles. However, studies on the use of fish-derived hydrolysates, proteins, and peptides as MMP inhibitors and their applications for cosmeceuticals are limited. Only a few studies focus on the MMP inhibitory activity of marine fish-derived peptides. Ryu et al. reported the isolation of novel peptides from seahorses that effectively increased collagen release through the suppression of collagenases 1 and 3 [111]. The same group isolated a protein from seahorse with an ability to inhibit MMP-1, MMP-3, and MMP-13 [112]. Shen et al. reported the hydrolysis of fish muscle from *Collichthys niveatus* using four commercial enzymes, namely alcalase, neutrase, protamex, and flavourzyme to isolate the peptides [113]. The major amino acids observed in the hydrolysate were threonine, glutamic acid, phenylalanine, tryptophan, and lysine. The total content of essential amino acids was calculated to be 970.7 ng/mL. The study was performed to check the effects of enzymatic hydrolysis conditions on the composition and properties of the peptides obtained from the hydrolysate, which could be utilized as health supplements [113]. A proteinase inhibitor (21 kDa) with similar properties to human tissue inhibitor of MMP-2 (TIMP-2) was obtained from Atlantic cod muscle and then identified by using gelatin affinity chromatography, real-time reverse zymography, and mass spectroscopy [114]. The amino acid sequences of the two peptides obtained from the inhibitor showed a high similarity to those of the human TIMP-2. The inhibitor was found to inhibit the gelatin-degrading enzymes.

## 5. Photo-Protective and Anti-Photoaging Activity of Fish Peptides and Fish Protein Hydrolysates

Skin is made up of three different layers, namely epidermis, dermis, and hypodermis. It acts as a chemical and physical barrier to protect the body against harmful foreign pollutants [115]. Skin can be damaged by various external environmental attacks, including harmful chemicals, ultraviolet (UV) light exposure, and temperature changes [116]. Photoaging and inflammation are often caused by UV radiation. Photoaging, also known as dermatoheliosis, is characterized by changes in the skin due to exposure of UV-A (400 to 320-nm wavelength) and UV-B (320 to 290-nm wavelength) light, which is main light source for photoaging [117]. The UV-A can permeate more deeply into the dermal matrix than UV-B, whereas UV-B is more carcinogenic compared to UV-A [118]. Considerable attention has been given to the utilization of marine fish-derived peptides for skin protection due to an excellent bioactivity, biocompatibility, penetration ability, and skin-repairing ability. Various fish-derived proteins and peptides have been investigated for their usage in the protection of skin from UV exposure [119,120,121].

Fish skin collagen and hydrolysates demonstrated a high biocompatibility with an ability to provide protection against the detrimental effects of UV radiation (Table 6). Zhuang et al. reported that jellyfish (*Rhopilema esculentum*) collagen (JC) and jellyfish collagen hydrolysate (JCH) alleviated UV-induced abnormal changes of antioxidant defense systems such as superoxide dismutase and glutathione peroxidase [116]. Both JC and JCH significantly protected the skin lipid and collagen from UV radiation. In addition, the UV-induced changes in the total ceramide and glycosaminoglycans in the skin were recovered, thus maintaining the balance of lipid compositions in the skin. The mechanism is mainly based on the antioxidative properties of the both JC and JCH along with stimulation of skin collagen synthesis. The study indicated that JCH that has lower molecular weights as compared to JC provides a much stronger protection against UV-induced photoaging [116]. The importance of jellyfish collagen on the antioxidant activities is further strengthened by another study that reports jellyfish as an abundant source of collagen with a high potential for nutraceutical applications [122]. The effects of JC and JCH on UV-induced skin damage of mice were evaluated by the analysis of skin moisture as well as microscopic analyses of skin and immunity indexes [123]. It was observed that the moisture retention ability of UV-induced mice skin increased upon treatment with JC and JCH. Further histological analysis demonstrated that JC and JCH could repair the endogenous collagen and elastin protein fibers, thus maintaining the natural ratio of type I to type III collagen. The immunity indexes showed that JC and JCH played a pivotal role in enhancing the immunity of photoaging mice in vivo. Again, as mentioned above, JCH exhibited a much higher protective ability than JC [123].

**Figure 4:**
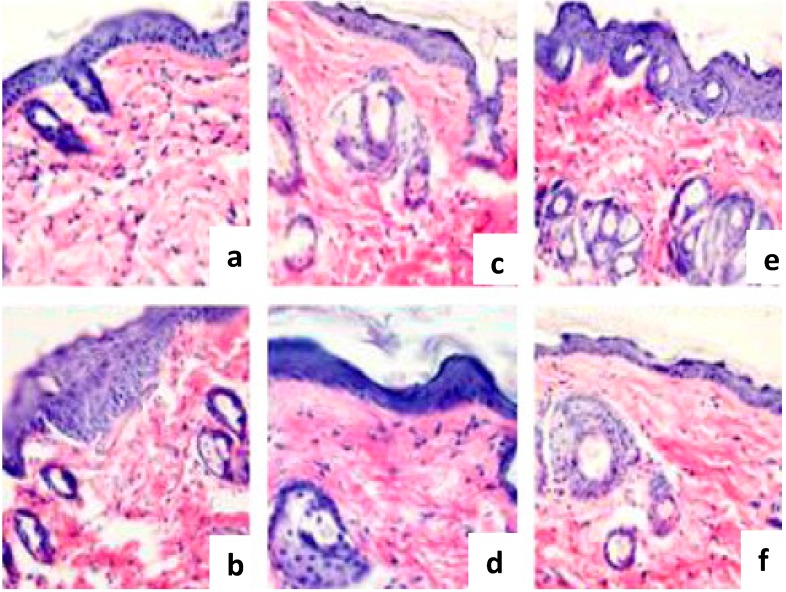
Effects of collagen polypeptide 1 and collagen polypeptide 2 on the morphology of photoaging skin (magnification 200×). (**a**) normal; (**b**) model; (**c**) collagen polypeptide 1 (50 mg/kg); (**d**) collagen polypeptide 1 (200 mg/kg); (**e**) collagen polypeptide 2 (50 mg/kg); and (**f**) collagen polypeptide 2 (200 mg/kg). Adapted with permission from [124].

Hou et al. evaluated the effects of collagen polypeptides isolated from cod skin on UV-induced damage to mouse skin [124]. Collagen polypeptide fractions (CP1 (2 kDa < Mr < 6 kDa) and CP2 (Mr < 2 kDa)) were obtained through pepsin digestion and alkaline protease hydrolysis methods. Collagen polypeptides provided good moisture absorption and retention properties, and CP2 was more efficient than CP1. In vivo studies demonstrated that both of the peptides provided protective effects against UV-induced wrinkle formation and destruction of skin structures (Figure 4). The action mechanisms of the collagen polypeptides mainly involve increasing immunity, decreasing the loss of moisture and lipid, and repairing endogenous collagen and elastin protein fibers [124].

Chen et al. studied the effects of gelatin hydrolysate extracted from the Pacific cod (*Gadus macrocephalus*) skin on UV radiation-induced inflammation and collagen reduction in photoaging mouse skin. Oral administration of gelatin hydrolysate suppressed UV radiation-induced damage to the skin by inhibiting the depletion of endogenous antioxidant enzyme activity, and by suppressing the expression of nuclear factor-κB (NF-κB) as well as NF-κB-mediated expression of pro-inflammatory cytokines. Furthermore, gelatin hydrolysate inhibited type I procollagen synthesis by up-regulating the type II transforming growth factor β (TGFβ) receptor (TβRII) level and down-regulating Smad7 levels, which demonstrates that gelatin hydrolysate is involved in matrix collagen synthesis by activating the TGF-β/Smad pathway in the photoaging skin [125].

Age-related skin thinning is involved in a decrease in the content of collagen in the skin. Co-treatment with collagen peptide and vitamin C upregulates the type I collagen in vivo. Shibuya et al. demonstrated that the collagen peptides supplemented with vitamin C reduced the superoxide dismutase 1 (Sod-1) [126]. In vitro studies further revealed that collagen oligopeptide, a digestive product of ingested collagen peptide, significantly enhanced the bioactivity of the vitamin C derivative with respect to the migration and proliferation of fibroblasts [126]. The collagen peptide and the vitamin C derivative additively increased the skin thickness of hairless Sod1-deficient mice.

Recently, gelatin and its hydrolysates from salmon skin were used to protect the skin from photoaging [127]. The average molecular weights of the gelatin and gelatin hydrolysates were found to be 65 kDa and 873 kDa, respectively [127]. In another study, dose effects of orally administered collagen hydrolysates on the UV-B-irradiated skin damage were investigated using UV-B-irradiated hairless mice [128]. The low dose of collagen hydrolysates increased the skin hydration and reduced the transepidermal water loss in the damaged skin [128]. In addition to this, tilapia gelatin peptides were investigated against UV-induced damage to mouse skin [129]. The results suggested that tilapia gelatin had an ability to avoid the UV damage by protecting the collagen and lipid in the skin. The antioxidant peptide, Leu-Ser-Gly-Tyr-Gly-Pro (592.26 Da), was identified from the tilapia gelatin peptides, and the peptide has an ability to scavenge the hydroxyl radicals with the IC_50_ value of 22.47 µg/mL [129,130].

**Figure 5:**
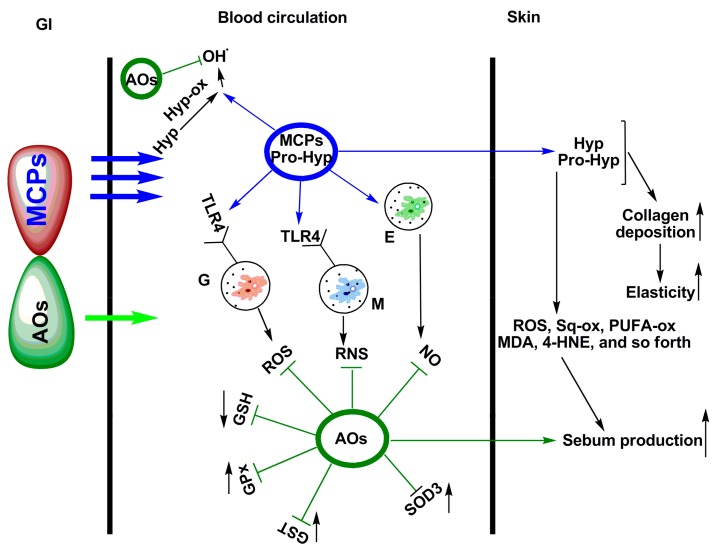
Scheme of the hypothesized redox-dependent mechanisms of physiological effects after co-treatment of marine collagen peptides (MCPs) and skin-targeting antioxidants (AOs). Redrawn with permission from [131]. In the figure, the three arrows (blue) indicate that MCPs easily penetrate the gastrointestinal wall (GI) through blood circulation and are mainly deposited in the skin. The single arrow (green) indicates that AOs are partially metabolized. However, AOs can reach the different layers of skin. While circulating in the blood, MCPs activate blood phagocytes (i.e., granulocytes (G) and monocytes (M)) and endotheliocytes (E) to generate reactive oxygen species (ROS) and reactive nitrogen species (RNS) by provoking Toll-like receptor-4 (TLR4)-mediated signals. Co-administered antioxidants can prevent systemic oxidative stress by blocking glutathione (GSH) oxidation, and activation of glutathione peroxidase (GPx), glutathione-S-transferase (GST), and superoxide dismutase 3 (SOD3).

The major concern regarding the safety and clinical feasibility of administration of marine collagen peptides (MCPs) has been raised because MCPs from different origin can activate innate immune response through Toll-like receptor 4 (TLR4)-mediated NADPH-oxidase (NOX4) activation and over-production of reactive oxygen species (ROS) [131]. Figure 5 represents the hypothesized redox-dependent mechanisms behind the physiological effects of fish skin MCPs combined with plant-derived skin-targeting antioxidants (coenzyme Q10 + grape-skin extract + luteolin + selenium) [131]. The MCPs were derived from the skin of deep sea fish (e.g., *Pollachius virens, Hippoglossus hippoglossus, and Pleuronectes platessa*). MCPs easily penetrate the gastrointestinal (GI) wall (three arrows) through blood circulation and are mainly deposited in the skin [131]. The clinical study demonstrated that combination treatment of MCPs with skin-targeting antioxidants can remarkably improve skin elasticity and sebum production while lowering the oxidative damage [131]. These results clearly indicate that skin-targeting antioxidants are essential components of MCPs-containing cosmeceuticals for more effective and safe treatment.

## 6. Conclusions

Marine fish-derived proteins and peptides are becoming the important resource for cosmetic industries. Several bioactive proteins and peptides were produced from marine fish via chemical or enzymatical hydrolysis and regarded as a safer option for the development of cosmeceutical products. The use of marine fish-derived proteins and peptides contribute to alleviating the environmental pollution caused by the waste generated by fish processing industries. Much attention has been paid to marine fish collagen for cosmeceutical applications owing its properties for skin hydration, with low odor and improved mechanical strength. In addition, marine fish-derived peptides have been extensively explored for cosmeceutical applications due to their various biological properties including antioxidant, antimicrobial, MMP inhibitory, photo-protective, and anti-photoaging activities. These biological activities of the marine fish peptides have led to the development of several types of anti-aging, skin care, and anti-wrinkle products. Despite the great potential of marine fish-derived proteins and peptides for cosmeceutical applications, most of them are still in the experimental stage and need to be further investigated with regard to their formulations and long-term safety for successful commercialization. Moreover, development of supplements that can further increase the bioavailability and tissue regeneration efficacy of marine fish-derived proteins and peptides is also required to increase their potential for cosmeceuticals. 

## Figures and Tables

**Table 1:** Important marine fish species used to isolate collagen.

Fish Species Name	Parts	Method	Yield (%)	Reference
*Lagocephalus gloveri*	Skin	PSC	54.3	[17]
*Thunnus obesus*	Bone	ASC and PSC	--	[19]
*Paralichthys olivaceus, Sebastes schlegeli, Lateolabrax maculatus, Pagrus major*	Skin	ASC	--	[22]
*Takifugu rubripes*	Skin	ASC and PSC	10.7 and 44.7	[27]
*Sepiella inermis*	Skin	ASC and PSC	0.58 and 16.23	[28]
*Lutjanus vitta*	Skin	ASC and PSC	9.0 and 4.7	[29]
*Magalaspis cordyla*	Bone	ASC and PSC	30.5 and 27.6	[30]
*Otolithes ruber*	Bone	ASC and PSC	45.1 and 48.6	[30]
*Evenchelys macrura*	Skin	ASC and PSC	80 and 7.1	[31,32]
*Saurida* spp., *Trachurus japonicus, Mugil cephalis, Cypselurus melanurus, Dentex tumifrons*	Scales	ASC	0.13–1.5%	[33]
*Cyanea nozakii* Kishinouye	All parts	ASC and PSC	13.0 and 5.5	[34]
*Sardinella longiceps*	Scales	ASC and PSC	1.25 and 3	[35]
*Priacanthus tayenus*	Skin and bone	ASC	10.94 and 1.59 (Skin and bone)	[36]
*Priacanthus tayenus*	Skin	PSC	--	[37]
*Priacanthus tayenus and Priacanthus macracanthus*	Skin	PSC	--	[38]
*Parupeneus heptacanthus*	Scale	ASC and PSC	0.46 and 1.2	[39]
*Mystus macropterus*	Skin	ASC and PSC	16.8 and 28	[40]
*Syngnathus schlegeli*	All parts	ASC and PSC	5.5 and 33.2	[41]
*Jellyfish*	All parts	PSC	46.4	[42]
*Chrysaora* sp.	All parts	PSC	9–19	[43]

**Table 2:** Conditions for the enzymatic hydrolysis of tuna backbone proteins.

Enzymes for Hydrolysis	Buffer	pH	Temperature (°C)
alcalase	0.1 M Na_2_HPO_4_–NaH_2_PO_4_	7	50
α-chymotrypsin	0.1 M Na_2_HPO_4_–NaH_2_PO_4_	8	37
papain	0.1 M Na_2_HPO_4_–NaH_2_PO_4_	6	37
pepsin	0.1 M Glycine–HCl	2	37
neutrase	0.1 M Na_2_HPO_4_–NaH_2_PO_4_	8	50
trypsin	0.1 M Na_2_HPO_4_–NaH_2_PO_4_	8	37

**Table 3:** Biological activities for cosmeceutical applications. MMP: matrix metalloproteinase.

Activity	Cosmeceutical Applications	Reference
Antioxidant	Anti-aging, photo-protective effects	[15]
Tyrosinase inhibitor	Whitening	[75]
MMP inhibitor	Anti-wrinkle	[76]
Anti-inflammatory	Skin soothing	[77]

**Table 4:** Potential bioactive antioxidant peptides from marine fish resources.

Fish Species Name	Enzymes for Hydrolysis	Peptides (Amino Acid Sequence)	Reference
*Scomber austriasicus*	protease N	--	[60]
*Thunnus obesus*	alcalase, α-chymotrypsin, neutrase, papain, pepsin, and trypsin	H-Leu-Asn-Leu-Pro-Thr-Ala-Val-Tyr-Met-Val-Thr-OH	[71]
Salmon	alcalase, flavourzyme, neutrase, pepsin, protamex, and trypsin	Peptides (unknown sequence, 1000–2000 Da)	[86]
*Decapterus maruadsi*	alcalase, neutral protease, papain, pepsin, and trypsin	His-Asp-His-Pro-Val-Cys and His-Glu-Lys-Val-Cys	[87]
*Johnius belengerii*	pepsin, trypsin, papain, α-chymotrypsin, alcalase, and neutrase	Glu-Ser-Thr-Val-Pro-Glu-Arg-Thr-His-Pro-Ala-Cys-Pro-Asp-Phe-Asn	[88]
*Paralichthys olivaceus*	papain, pepsin, trypsin, neutrase, alcalase, kojizyme, protamex, and α-chymotrypsin	Val-Cys-Ser-Val and Cys-Ala-Ala-Pro	[89]
*Magalaspis cordyla*	pepsin, trypsin, and α-chymotrypsin	Ala–Cys–Phe–Leu (518.5 Da),	[90]
*Magalaspis cordyla*	pepsin/trypsin, and α-chymotrypsin	Asn-His-Arg-Tyr-Asp-Arg (856 Da)	[91]
*Otolithes ruber*	pepsin/trypsin and α-chymotrypsin	Gly-Asn-Arg-Gly-Phe-Ala-Cys-Arg-His-Ala (1101.5 Da)	[91]
*Johnius belengerii*	trypsin, R-chymotrypsin, and pepsin	His-Gly-Pro-Leu-Gly-Pro-Leu	[92]
*Otolithes ruber*	pepsin, trypsin, and α-chymotrypsin	Lys-Thr-Phe-Cys-Gly-Arg-His	[93]
*Oreochromis niloticus*	alcalase, pronase E, pepsin, and trypsin	Asp-Pro-Ala-Leu-Ala-Thr-Glu-Pro-Asp-Pro-Met-Pro-Phe	[94]
*Merluccius productus*	Validase ^®^ BNP (V) and Flavourzyme ^®^	--	[95]
*Oreochromis niloticus*	properase E and multifect neutral	Glu-Gly-Leu (317.33 Da) and Tyr-Gly-Asp-Glu-Tyr	[96]
*Hypoptychus dybowskii*	alcalase, neutrase, α-chymotrypsin, papain, pepsin, and trypsin	Ile–Val–Gly–Gly–Phe–Pro–His–Tyr–Leu	[97]

**Table 5:** Marine fish species and enzymes used in the isolation of antimicrobial peptides. Targeted microorganisms used to check the marine fish-derived antimicrobial peptides are shown.

Name of Fish Species	Enzymes for Hydrolysis	Microorganisms	Reference
*Setipinna taty*	pepsin	*Escherichia coli*	[107]
*Setipinna taty*	papain, pepsin, trypsin, alkaline protease, acidic protease, and flavoring protease	*Escherichia coli, Pseudomonas fluorescens Proteus vulgaris, Bacillus megaterium Staphylococcus aureus, Bacillus subtilis, Bacillus megaterium, Sarcina lutea*	[108]
*Scomber scombrus*	--	*Listeria innocua, Escherichia coli*	[109]
*Scomber scombrus*	protamex, neutrase, papain, and flavourzyme.	*Listeria innocua HPB13 and Escherichia coli*	[110]

**Table 6:** Photo-protective and anti-photoaging proteins and peptides from marine fish.

Name of Fish Species and Parts	Fish-Derived Proteins and Peptides	Enzymes for Hydrolysis	Reference
Jellyfish	Collagen	properase E	[123]
Cod skin	Collagen polypeptides	alkaline protease and pepsin	[124]
Cod skin	Gelatin hydrolysate	alkaline protease and trypsin	[125]
Salmon skin	Gelatin	alkaline protease and trypsin	[127]
Tilapia	Gelatin peptides	properase E	[129]
*Pollachius virens, Hippoglossus hippoglossus, and Pleuronectes platessa*	Marine collagen peptides	complex proteases	[131]
